# miRNAs in Adipocyte-Derived Extracellular Vesicles: Multiple Roles in Development of Obesity-Associated Disease

**DOI:** 10.3389/fmolb.2020.00171

**Published:** 2020-07-29

**Authors:** Yaliang Zhou, Chunlu Tan

**Affiliations:** Department of Pancreatic Surgery, West China Hospital, Sichuan University, Chengdu, China

**Keywords:** miRNAs, extracellular vesicles, adipose, obesity, obesity-associated disease

## Abstract

Obesity and overweight are common modern health challenges. Caloric intake greater than that needed for energy production results in excess storage of fat in the abdomen. Visceral fat secretes a wide spectrum of adipokines, and increased adiposity is associated with a higher risk of development of metabolic disorders. In addition, adipose tissue secretes extracellular vesicles (EVs) to communicate with peripheral cells and distant organs, and regulate whole-body metabolism. Furthermore, clinical evidence has shown that adipose tissue-derived EVs are present at low levels in the circulation of healthy individuals. In contrast, individuals with metabolic syndrome have significantly higher levels of circulating adipose-derived EVs. The composition of the contents of EVs is dynamic, and closely mirrors individual daily habits and fasting-fed state metabolic characteristics. In this mini-review, we aimed to elucidate the role of adipocyte-derived EVs in regulation of whole-body metabolism under physiological and pathophysiological conditions. Studies have shown that adipose tissue may be a major source of circulating exosomal miRNAs that regulate metabolic homeostasis and directly promote insulin-resistance in other organs. Furthermore, the composition of adipocyte-derived circulating miRNAs in EVs may change prior to development of metabolic disorder. Adipocyte-derived miRNAs in EVs may also induce obesity-related changes such as M1 polarization and inhibition of M2 polarization of macrophages, which may affect the biological behaviors of surrounding tumor cells.

## Introduction

Obesity and overweight are serious public health concerns linked to complications such as cardiovascular disease (Dwivedi et al., 2020), type 2 diabetes (Zhang T. et al., 2019), hypertension (Nguyen et al., 2008), and many types of cancers (Renehan et al., 2008). Besides a wide spectrum of adipokines, adipose tissue may secrete extracellular vesicles (EVs) as a means of communication with peripheral cells (Xie et al., 2018) and distant organs (Dang et al., 2019) to regulate whole-body metabolism under both physiological and pathophysiological conditions.

Extracellular vesicles are membrane structures released by cells that play a role to exchange components between cells. Based on the current research those were comprehended by transmission and immuno-electron microscopy and by biochemical means, EVs typically are classified into microvesicles and exosomes.

Exosomes are 30–150 nm in diameter, generated through inward budding of endosomal membranes which leads to form multivesicular endosomes (MVEs) (Harding et al., 1984). The cargoes in exosome including intracellular proteins, lipids, RNA, and DNA are influenced by the physiological or pathological state of the donor cell. Microvesicles are 100–1,000 nm in diameter and originate at the plasma membrane by an outward budding and fission of the plasma membrane. However, sometimes it is hard to differentiate microvesicles from exosomes because of the overlapping range of size, similar morphology, and similar cargoes.

Multiple reports have implicated EVs as a vehicle transferring miRNAs between cells (Dang et al., 2019; Li et al., 2019). Studies have shown that metabolic diseases affect the total amount of vesicle-transported miRNAs in circulation. Circulating EV number was found higher in individuals with impaired oral glucose tolerance test (OGTT) (Kobayashi et al., 2018). Exosomal miRNAs such as miR-23b, miR-148b, miR-4269, and miR-4429 derived from obese visceral adipose expressed differently comparing to lean visceral adipose in adolescent, and TGF-β and Wnt/β-catenin signaling emerged as top canonical pathways targeted by these miRNAs (Ferrante et al., 2015). Santamaria-Martos et al. (2020) identified some EV-contained miRNA candidates related to BMI (e.g., let-7b, miR-146a) and fasting insulin (e.g., miR-222/223, miR-26b) in the plasma of obese and non-obese women who were absent of acute or systemic disease. This review focuses on the diverse roles of miRNAs packaged within adipocyte-derived EVs in the development of obesity-related disease.

## Circulating Adipocyte-Derived EVs Are Associated With Clinical Parameters of Metabolic Syndrome

The prevalence of metabolic syndrome is directly associated with the incidence of being overweight or obese (Wang et al., 2011). Diagnosis of metabolic syndrome is based on BMI, blood pressure, blood glucose levels, and blood lipid biochemical indices. However, there are no sensitive clinical indicators that allow intervention prior to development of pathological metabolic disorders. Recent studies have shown that the number of EVs circulating in the blood is closely related to metabolic status, and EVs may be sensitive indicators of metabolic disorders (Agouni et al., 2014; Kobayashi et al., 2018).

In healthy individuals, EVs primarily derive from platelets, smaller population derive from monocytes, and vascular endothelial cells (Christersson et al., 2013). Connolly et al. showed that when EVs from cells of blood and vessels that might contain adipocyte markers and adiponectin were depleting, adipocyte markers were still detectable in plasma EV samples. This finding indicated that some EVs in the circulation system may have originated from adipocytes (Connolly et al., 2018).

Large numbers of non-circulation-derived EVs may indicate a pathological state. Eguchi et al. showed that obese individuals had greater levels of circulating EVs than lean individuals. Moreover, the abundance of perilipin A in circulating EVs of obese individuals was higher than that of lean individuals, which suggested that these EVs might have originated in adipose tissue (Eguchi et al., 2016). However, perilipin A was not detected in circulating EVs in healthy obese individuals in previous studies (Bastos-Amador et al., 2012; Ostergaard et al., 2012). Administration of a 1,500-kcal/day caloric restriction dietary intervention for 3 months to obese individuals with metabolic syndrome reduced the number of circulating EVs, BMI, and fat mass. Moreover, changes in the numbers of circulating EVs and levels of perilipin A in circulating EVs were significantly positively correlated with changes in insulin levels and HOMA-IR values (Eguchi et al., 2016). These results suggested that the numbers of circulating EVs and perilipin A levels were more closely related to metabolic stress than BMI or fat mass.

Kobayashi et al. analyzed circulating EVs in 203 participants with or without risk factors for metabolic diseases. The results showed that the number of circulating EVs correlated most strongly with risk factors for metabolic diseases. They further isolated circulating EVs and found that perilipin A was correlated with serum triglyceride (TG) levels, asialoglycoprotein receptor 1 (ASGPR1) levels were significantly increased in individuals with metabolic risk factors. In contrast, levels of annexin V and CD9, which are common EV markers, did not differ between individuals with or without metabolic risk factors (Kobayashi et al., 2018). Perilipin A is enriched in adipose tissue (Greenberg et al., 1991) and steatotic hepatocytes (Straub et al., 2008) and ASGPR1 is enriched in hepatocytes (Matsuura et al., 1982), which suggests that the levels of adipocyte- and hepatocyte-derived EVs may be increased in individuals experiencing metabolic stress.

## Complex Relationship Between Dietary Habits and Adipocyte-Derived EVs

Obesity occurs as a result of increased food intake or decreased metabolism. Adipose tissue is critical to systemic energy homeostasis because it participates in the regulation of metabolic organs. Previous studies have described how the functions of adipocyte-derived EVs are integrated and coordinated in accordance with system-wide metabolic demands.

### The Contents of Adipocyte-Derived EVs Differ Between Fasted and Fed States

Clair et al. found that fasting enhanced the abundance of signaling pathways in adipose tissue secreting EVs compared to the fed stage. For example, proteins involved in Wnt signaling and in the mitochondrial respiratory chain were increased in the fasted state. Adipocytes may receive EVs that contain membrane proteins from endothelial cells and then transport these proteins into adipocyte-derived EVs. Glucagon signaling enhanced endothelial cell uptake and packaging of extracellular cargos into EVs without enhancing endothelial cell EV secretion in mice fed a high-fat diet (Crewe et al., 2018).

### Adipocyte-Derived EVs May Modulate Appetite and Weight

Gao et al. showed that adipocyte-derived EV-associated MALAT1 increased mTOR expression and decreased POMC expression in hypothalamic POMC neurons. *In vitro* results showed that EV−associated MALAT1, which was significantly increased in adipocytes of obese mice, regulated mTOR signaling through miR−181b and miR−144. Adipocyte-derived EVs from obese mice increased appetite and weight gain in lean mice. In contrast, adipocyte−derived EVs from lean mice attenuated appetite and reduced body weight of obese mice (Gao et al., 2019).

## The Importance of miRNAs in Circulating Adipocyte-Derived EVs

### Adipose Tissue May Be a Major Source of Circulating Exosomal miRNAs

The majority of the circulating EVs in the plasma of healthy individuals originate from blood and vessel cells. Adipocyte-derived EVs may constitute a relatively small fraction of the total EV population in circulating plasma. However, this may not directly reflect potential effects of adipocyte-derived EVs on an individual’s health, particularly with regard to EVs derived from dysfunctional adipocytes. The bioactive molecules transported in adipocyte-derived EVs are likely to exert their functions in the circulation. In an individual suffering from metabolic stress, increased numbers of EVs may further enhance the effects of their contents. Although “classical” adipokines such as adiponectin, leptin, and fatty acid binding protein 4 (FABP4) have been identified in adipocyte-derived EVs (Phoonsawat et al., 2014; Connolly et al., 2015), these proteins in EVs comprise a small fraction of all in the total circulating level. These proteins in EVs may play a more significant role in cross-talk between adipocytes and surrounding cells than in modulation of distant targets.

miRNAs in adipocyte-derived EVs may play a more important role in the circulation than proteins such as leptin, adiponectin, or FABP4. Thomou found that the levels of 422 of 653 detectable exosomal miRNAs were altered in the serum of the mice with an adipose-tissue-specific knockout of the miRNA-processing enzyme Dicer (ADicerKO) which exhibits a defect in miRNA processing in adipose tissue, comparing with the wild type. The circulating levels of exosomal miRNAs were rescued following transplantation of brown adipose tissue (BAT) into the ADicerKO mice. In addition, Thomou showed that lipodystrophic patients exhibited drastically reduced levels of exosomal miRNAs in the bloodstream compared to those in controls. However, the numbers of exosomes did not differ between these two groups. This finding suggested that reduced levels of miRNAs were due to altered miRNA processing in adipocytes, and not the number of circulating exosomes (Thomou et al., 2017). These results indicated that adipose tissue was a major source of circulating exosomal miRNAs.

### Circulating miRNAs in EVs Could Regulate Metabolic Homeostasis

Conventional adipokines and proteins with catalytic activities may be secreted by adipocytes into the circulation as a mechanism to regulate metabolic homeostasis (Barzilai et al., 1997; Yamauchi et al., 2001). Exosomal miRNAs may also act as endocrine effectors to regulate metabolic homeostasis *in vivo*. Lean mice that were administered adipose tissue macrophage (ATM)-derived exosomes with increased levels of miR-155 from wild type obese mice by high-fat feeding were found to exhibit glucose intolerance and insulin resistance (Ying et al., 2017). Thomou et al. transplanted BAT from normal mice into ADicerKO mice, which resulted in restoration of circulating exosomal miRNA levels, and improved glucose tolerance and insulin sensitivity. However, serum levels of traditional adipokines such as leptin, adiponectin, and serum IL-6 were not restored, which indicated that the observed improvements may have been dependent on restoration of the circulating exosomal miRNA levels (Thomou et al., 2017).

### miRNAs in Circulating Adipocyte-Derived EVs Regulate Gene Expression in Other Tissues

Thomou found that miR-99b in adipocyte-derived EVs reduced Fgf21 mRNA levels in the liver. In addition, regulation of FGF21 only occurred through exosomal delivery of miR-99b, and not in response to direct incubation with miR-99b. Transfection of mouse BAT with an adenovirus containing the human-specific miRNA hsa_miR-302f resulted in targeting of exosomes in the circulation to an hsa_miR-302f 3′ UTR reporter present in the liver (Thomou et al., 2017). This finding suggested that miRNAs in adipocyte-derived EVs may play an endocrine role similar to that of traditional adipokines.

### Circulating miRNAs in EVs Directly Induce Insulin-Resistance in Other Organs

Recent studies have shown that adipocyte-derived EVs in mice promoted systemic insulin resistance. Dang et al. showed that adipocyte-derived exosomes from obese and high fat-fed mice inhibited glucose uptake and promoted insulin resistance in AML12 hepatocytes. miR-141-3p expression was decreased in AML12 cells co-cultured with exosomes from obese mice *in vitro*, and in liver tissue of obese mice. Furthermore, this study showed that exosomes released from obese adipose tissue transferred less miR-141-3p into hepatocytes than exosomes from lean adipose tissue, which resulted in inhibition of hepatocyte glucose uptake and induction of insulin resistance (Dang et al., 2019). Yu et al. showed that miR-27a expression was increased in visceral adipose tissue of mice fed a high-fat diet. Furthermore, miR-27a was secreted into the circulation by adipocytes in exosomes, and then taken up by skeletal muscle cells. Accumulation of mirR-27a in skeletal muscle cells repressed PPARγ, which resulted in impaired insulin signaling (Yu et al., 2018).

### Levels of Circulating miRNAs in Adipocyte-Derived EVs Change Prior to Onset of Metabolic Disorder

Levels of circulating miRNAs in adipocyte-derived EVs depend on individual environmental factors and adipocyte status. Chen et al. found that circulating miR-92a was downregulated in exosomes derived from BAT and inguinal white adipose tissue (WAT) of mice exposed to cold stimulation. In addition, the exposure of 10 people to a 10-day cold acclimation period resulted in decreased levels of miR-92a (Chen et al., 2016).

Daily dietary habits such as different glycemic load dietaries, and physical activities such as acute or chronic exercise also induce changes in the expression levels of miRNAs in the circulation (McCann et al., 2013; Nielsen et al., 2014). Several mechanisms may be responsible for the changes. One mechanism may be reduction in secretion from original cells in the form of exosomes and lipoproteins, another possibility is that degradation of circulating miRNAs is accelerated or uptook from circulation into certain recipient cells. Many of these miRNAs are expressed in adipose tissue; some of these miRNAs, such as miR-92a and miR-193b, are differentially expressed in obese and healthy individuals (Arner and Kulyte, 2015). These differences in miRNAs in adipose tissue may lead to variations in the levels of these miRNAs in exosomes, which may alter the effects of these exosomes on other tissues. Therefore, it is important to confirm the relationship between changes in circulating miRNAs in EVs and obesity-related diseases to determine whether these changes in circulating miRNAs are a cause or effect of disease states.

Hubal et al. evaluated global miRNA profiles of circulating adipocyte-derived exosomes before and 1 year after gastric bypass surgery of obese patients. The results showed that changes in 46 known mature miRNAs correlated with changes in HOMA (Hubal et al., 2017). This finding indicated that circulating miRNAs in adipocyte-derived EVs were closely related to improvements in pathological metabolism in obese patients. Thomou found that the expression levels of 225 miRNAs were altered in circulating adipocyte-derived EVs of young ADicerKO mice. Young ADiceKO mice exhibit metabolic phenotypes similar to those of wild-type mice at the same age (Thomou et al., 2017). This finding indicated that changes in levels of circulating adipocyte-derived exosomal miRNAs may cause, and not be a consequence of, metabolic disorder. Furthermore, this study provided evidence for the importance of miRNAs incorporated in circulating adipocyte-derived EVs *in vivo*.

## Cross-Talk Between miRNAs in Adipocyte-Derived EVs and Macrophages

Obesity is closely related to chronic inflammation, as evidenced by the accumulation of immune cells in adipose tissue. Recent studies found that adipocyte-derived EVs play an important role in macrophage infiltration. For example, exosomes derived from visceral adipose tissue of high fat-fed animals promoted macrophage M1 polarization through activation of NF-κB (Xie et al., 2018).

### MiRNAs in Adipocyte-Derived EVs Mediate M1 Macrophage Polarization

Obesity induces accumulation of M1 polarized macrophages in adipose tissue. Studies have shown that M1 polarization of ATMs in obesity is associated with inflammation and insulin resistance in adipose tissue (Lumeng J. L. et al., 2007; Fujisaka et al., 2011). Exosomes released from adipose tissue stimulate peripheral blood monocytes to differentiate into activated macrophages with increased secretion of TNF-α and IL-6 (Deng et al., 2009). Zhang et al. (2016) showed that miR-155 could be delivered to bone marrow-derived macrophages by adipocyte-derived exosomes, which resulted in targeting of SOCS1 and modulation of M1 macrophage polarization via JAK/STAT signaling.

### miRNAs in Adipocyte-Derived EVs Inhibit M2 Macrophage Polarization

Lean adipose tissue contains predominantly M2-polarized macrophages, which play a key role in maintaining homeostasis. However, during the development of obesity, ATMs populated in adipose tissue shift from anti-inflammatory M2 to proinflammatory M1, which results in production of proinflammatory factors that exacerbate insulin resistance (Lumeng S. M. et al., 2007; Lumeng et al., 2008). Pan et al. (2019) demonstrated that miR-34a in adipocyte-derived exosomes could be transported into macrophages, resulting in inhibition of M2 polarization through inhibition of the expression of Krüppel-like factor 4. In addition, the expression of miR-34a was reportedly increased in adipose tissue of obese humans (Ortega et al., 2010).

## Adipocyte-Derived EVs Act as an Intermediary Between Hormones and Other Cells

Adipose tissue is capable of producing and secreting a variety of adipokines and cytokines that regulate metabolic homeostasis. Adiponectin is a representative adipocytokine, which circulates at very high concentrations, constituting approximately 0.01–0.05% of total serum proteins. The secretion of adiponectin in the form of classical or/and exosome pathways by adipocyte is influenced by hormones (e.g., prolactin and growth hormone) (Nilsson et al., 2005) and metabolites in circulation (fatty acids) (DeClercq et al., 2015). Next, adiponectin accumulates in tissues such as heart, vascular endothelium, and skeletal muscles through interaction with T-cadherin, protecting against cellular damage (Denzel et al., 2010) and metabolic diseases (Holland et al., 2017), enhancing exosome biogenesis and release to decreases cellular ceramides (Obata et al., 2018).

On the other hand, adipocyte-derived EVs could also regulate or protect target cells or organs, responding to circular hormones. Melatonin administration has been shown to increase cellular α−ketoglutarate in adipocytes of obese mice and to increase levels of α−ketoglutarate in exosomes secreted by adipocytes. These exosomes then promote M2 macrophage activation through increased α−ketoglutarate levels in macrophages (Liu et al., 2018). Melatonin has also been shown to reduce resistin levels in adipocyte−derived exosomes ameliorate hepatic steatosis (Rong et al., 2019).

## miRNAs in EVs Promote Cancer Growth and Migration

Obesity may promote tumor development through enhanced secretion of proinflammatory molecules, altered energy metabolism, secretion of pro-angiogenic factors, and immune cell recruitment and macrophage polarization. Recent studies have shown that adipose-derived EVs influence the biological behavior of tumor cells through transport of circRNAs to hepatocellular carcinoma (Zhang A. et al., 2019) and MMP3 to lung cancer cells (Wang et al., 2017). miRNA 23a/b has been shown to be transported into hepatic cancer cells via adipocyte-derived exosomes, resulting in cancer cell growth and migration, and development of chemoresistance through targeting of the von Hippel-Lindau/hypoxia-inducible factor axis (Liu et al., 2019).

## Future Perspectives and Considerations

### Indicators for Sub-Health Monitoring

**FIGURE 1:**
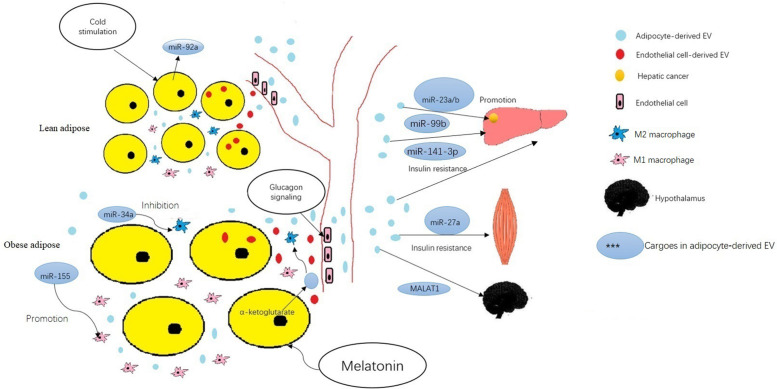
The roles of miRNAs and other cargoes mentioned in the text contained in adipocyte-derived EVs in regulating metabolism and development of obesity-associated disease. ^∗^The cargoes in adipocyte-derived EVs adjust in physiological and pathological states. Cold stimulation down regulates the circulating miR-92a in EVs of adipose tissue. Adipocytes may receive EVs that contain membrane proteins from endothelial cells, then transport these proteins into adipocyte-derived EVs. Glucagon signaling enhanced endothelial cell uptake and packaging of extracellular cargos into EVs in mice fed a high-fat diet. ^∗^Adipose−derived EVs mediate inter−organ cross-talk. The miR-99b in adipocyte-derived EVs regulated gene expression in the liver. Adipocyte-derived EV-associated MALAT1 regulated signaling in hypothalamic POMC neurons. In obesity, miR-141-3p in adipocyte-derived EVs promoted insulin resistance in hepatocytes and miR-27a in adipocyte-derived EVs promoted insulin resistance in skeletal muscle cells. ^∗^Adipose−derived EVs mediate inter−organ cross-talk. Adipose−derived EVs regulate intra−organ cross-talk between adipocytes and ATM. The miR-155 in adipocyte-derived EVs modulates of M1 macrophage polarization. The miR-34a in adipocyte-derived EVs inhibits of M2 polarization. The miRNA 23a/b in adipose−derived EVs promotes hepatic cancer cell growth, migration, and development of chemoresistance. ^∗^Adipocyte-derived EVs act as an intermediary between hormones and cells. Melatonin administration increases levels of α−ketoglutarate in EVs secreted by adipocytes; these EVs then promote M2 macrophage activation through increased α−ketoglutarate levels in macrophages.

Adipose tissue is thought to play an important role in regulation of endocrine function, and can modulate metabolism in other organs. Recent studies suggest that adipose cells regulate metabolism in liver, skeletal muscle, even tumors through release of exosomes into the circulatory system. Many studies have shown that the miRNA in exosomes changes significantly during metabolic disease development, may serve as ideal markers for disease diagnosis. The roles of miRNAs contained in adipocyte-derived EVs in regulating metabolism and the biological behavior of tumors, and the variation under metabolic stress are listed in Table 1 and summarized in Figure 1.

**TABLE 1:** Representative studies of obesity-related miRNAs in adipocyte-derived EVs.

Subject of study	Markers in EV	Potential targets/pathways	Roles in development of obesity-associated disease
Obese healthy adolescent (Ferrante et al., 2015)	miRNA	TGF-β, Wnt/β-catenin	Induced changes in cellular microenvironment of obese healthy individuals
Obese healthy women (Santamaria-Martos et al., 2020)	miRNA		Induced changes in plasma of obese healthy individuals
ADicerKO mice (Thomou et al., 2017)	miRNA	–	Major circulating miRNA source, and regulated distant organs
ob/ob mice (Dang et al., 2019)	miR-141-3p	–	Inhibited hepatocyte insulin sensitivity
db/db mice (Yu et al., 2018)	miR-27a	PPARγ	Impaired insulin signaling in skeletal muscle
Bone marrow-derived macrophages (Zhang et al., 2016)	miR-155	SOCS1	Controlled M1 macrophage polarization
Macrophages (Pan et al., 2019)	miR-34a	Klf4	Suppressed M2 polarization
Hepatocellular cancer (Liu et al., 2019)	miR-23a/b	VHL/HIF	Promoted cancer cell growth and migration

However, exosomes in the circulatory system may originate from many cell types. Adipose tissue is supposed a major source of circulating exosomal miRNAs, even to enhance secreting amount of EVs under metabolic stress. Therefore, monitoring adipocyte-derived miRNAs in circulatory exosomes may represent a novel approach to prevention and treatment of obesity-related diseases. Thomou have shown levels of miRNAs in circulating adipocyte-derived EVs change prior to the onset of metabolic disorder (Thomou et al., 2017). Global changes in circulating exosomal miRNAs are considered indicators of increased risk for development of obesity-related diseases, and specific changes in miRNA levels may enable the prediction of increased risk of development of specific diseases. miRNAs may be more sensitive and more accurate predictors than existing clinical biochemical indicators, with potential applications in measures aimed at the prevention of disease.

### Monitoring Indicators to Determine Safe Dosing of Hormone Drugs

Some hormone drugs, such as melatonin, alter the levels of specific proteins or miRNAs in fat cells. These changes result in changes in the levels of protein or miRNA in exosomes secreted by fat cells. Titration of hormone drug dosing may benefit from monitoring the levels of target proteins or miRNAs in circulating EVs. This may result in better disease control and reduced adverse reactions.

### Therapeutic Targets for Obesity-Related Diseases

Hoshino et al. (2015) suggest that exosomes target specific organs through various surface integrins. Therefore, exosomes may be engineered and loaded with miRNAs for the treatment of obesity-associated diseases such as fatty liver and diabetes mellitus. In order to ensure the safety and efficacy of exosome-based RNA carriers for disease treatment, disease-specific requirements for adequate distribution of therapeutic exosomal miRNAs in the body and levels of exosomal miRNA must be met.

## Author Contributions

YZ contributed to drafting the review. CT contributed to designing and composing the review. Both authors contributed to the article and approved the submitted version.

## Conflict of Interest

The authors declare that the research was conducted in the absence of any commercial or financial relationships that could be construed as a potential conflict of interest.
